# Novel Computed Tomography Angiography Parameter Is Associated with Low Cardiac Index in Patients with Chronic Thromboembolic Pulmonary Hypertension: A Retrospective Analysis

**DOI:** 10.3390/jcdd11090281

**Published:** 2024-09-07

**Authors:** Estefania Oliveros, Michel Ibrahim, Carlos Manuel Romero, Paul Navo, Patricia Otero Valdes, Yevgeniy Brailovsky, Amir Darki, Riyaz Bashir, Anjali Vaidya, Paul Forfia, Chandra Dass

**Affiliations:** 1 Heart and Vascular Institute, Temple University Hospital, Philadelphia, PA 19140, USA; 2 ChenMed Cardiovascular Care, Miami, FL 33169, USA; 3 Division of Cardiology, University of Pittsburgh Medical Center, Pittsburgh, PA 15219, USA; 4 Department of Radiology, Temple University Hospital, Philadelphia, PA 19140, USA; 5 Lewis Katz School of Medicine, Temple University, Philadelphia, PA 19140, USA; 6 Division of Cardiology, Jefferson Heart Institute, Sidney Kimmel School of Medicine, Thomas Jefferson University, Philadelphia, PA 19144, USA; 7 Division of Cardiovascular Disease, Loyola University Medical Center, Loyola Stritch School of Medicine, Loyola University Hospital, Chicago, IL 60611, USA

**Keywords:** chronic thromboembolic pulmonary hypertension, computed tomography pulmonary angiography, Hounsfield unit, right heart catheterization, cardiac index, pulmonary thromboendarterectomy

## Abstract

Chronic thromboembolic pulmonary hypertension (CTEPH) is a complication of incomplete resolution of acute pulmonary embolism. We hypothesize changes in CT Hounsfield Unit gradient (HU-Δ) created by the dispersion of IV contrast through the downstream blood pool correlate with cardiac index (CI). We sought to compare HU-Δ with invasively obtained CI. Methods: We completed a retrospective analysis of CTEPH patients in which individuals with low CI (<2.2-L/min/m^2^) were identified. Both absolute and fractional HU-Δ were derived from pulmonary CTA by subtracting the HU value of the left atrium (LA) and left ventricle (LV) from the main pulmonary artery (MPA) (absolute) and expressing them as a percentage of MPA-HU (fractional) on static axial images. These were compared between low and normal CI. Results: Of the 237 patients, 50.2% were female, 53.2% were White, 36.7% were Black. Hemodynamics were mean pulmonary artery (PA) pressure = 45.4 ± 11.2-mmHg, pulmonary vascular resistance = 9.2 ± 4.4-WU, CI = 2.05 ± 0.48-L/min/m^2^. There was a higher mean MPA-HU = 391.1 ± 113.6 than LA-HU = 251.6 ± 81. In patients with low CI, the HU-Δ was higher, HU-ΔMPA-LA was 148.9 ± 78.4 vs. 124.5 ± 77.2 (*p* = 0.02), and HU-ΔMPA-LV was 170.7 ± 87 vs. 140 ± 82 (*p* = 0.009). A HU-ΔMPA-LA = 118 had a sensitivity of 75.6% and specificity of 77% to detect low CI, AUC 0.61, *p* = 0.003. A HU-ΔPA-LV = 156 had a sensitivity of 77% and specificity of 53% to detect low CI, AUC = 0.62, *p* = 0.001. A fractional reduction HU-ΔMPA-LA of 35% had a sensitivity and specificity of 79% and 53%, respectively, to detect low CI (AUC 0.65, *p* < 0.001). A fractional reduction of the HU-ΔMPA-LV of 40% had a sensitivity and specificity of 80% and 55%, respectively, to detect low CI (AUC 0.65, *p* < 0.001). HU Δ were highly reproducible (Kappa = 0.9, *p* < 0.001, 95% CI 0.86–0.95). Conclusions: High HU Δ between MPA-LA and MPA-LV were associated with low CI in patients with CTEPH.

## 1. Introduction

Chronic thromboembolic pulmonary hypertension (CTEPH) is pulmonary hypertension (PH) attributed to post-thromboembolic fibrotic obstructions within the pulmonary artery [1]. It occurs as a severe and relatively uncommon complication after an acute pulmonary embolism (PE). PH, regardless of its etiology, is a life-threatening condition associated with increased mortality [2]. It is of utmost importance to diagnose and treat this early in order to prevent or delay the development of fatal complications.

Currently, there are three specific treatment modalities available: surgical pulmonary thromboendarterectomy (PTE), balloon pulmonary angioplasty (BPA), and pulmonary vasodilators [3]. The treatment decision is based on the localization of the pulmonary vascular obstruction and the surgical candidacy of the patient. PTE is the gold standard treatment for CTEPH and can be curative. Therefore, evaluation of CTEPH by referral centers is mandatory.

When evaluating patients with CTEPH, risk stratification is performed to determine the most appropriate therapeutic intervention. Echocardiogram Doppler and right heart catheterization (RHC) will provide the hemodynamic assessment. Whereas an anatomic evaluation of the clot burden will be obtained with the ventilation–perfusion (VQ) scan, catheter-based pulmonary angiograms, computed tomography angiography (CTA), and, less commonly, magnetic resonance angiography [1,4]. The use of CTA as a surrogate to estimate hemodynamics has been described in the field of acute PE [5]. In chronic PE, investigators have described other quantitative and qualitative techniques to correlate hemodynamic measurements pre- and post-PTE [6].

The Hounsfield unit (HU) is a measure of how much radiation is attenuated by different tissues, and it is used to distinguish between tissues of different densities as well as the degree of post-contrast enhancement, including blood [7]. The change of blood density in HUs is affected by many variables, including contrast medium delivery and patient-related factors. In a previous study, researchers explored the association of contrast kinetics with cardiac output in acute PE and found that CTA-derived HU gradient (Δ) is a simple and readily available marker that is inversely proportional to cardiac index (CI) [5]. Consequently, estimation of HU-Δ non-invasively through CTA could offer additional information for risk stratification and the management of patients with CTEPH.

We hypothesize that HU-Δ related to the downstream dispersion of IV contrast through the cardio-pulmonary vascular system will correlate with CI. The purpose of our study was to investigate the correlation between HU-Δ and the invasively measured CI in patients with CTEPH.

## 2. Materials and Methods

### 2.1. Patient Population

We performed a retrospective chart review of 237 CTEPH patients referred to Temple University Hospital’s (TUH) Heart and Vascular Institute Pulmonary Hypertension, Right Heart Failure, and Chronic Thromboembolic Pulmonary Hypertension (CTEPH) Program. The CTEPH patients were treated with PTE or BPA. We collected basic demographics, comorbidities, and echocardiographic data. All patients underwent invasive hemodynamic testing with RHC and imaging with pulmonary CTA as part of their initial evaluation before surgery or catheter-based interventions. No changes in PH medications were undertaken in between studies. Patients were divided into two groups based on invasively derived CI: low CI (<2.2 L/min/m^2^) and normal CI (≥2.2 L/min/m^2^). The CI cut-off was chosen based on the recently published paper on acute PE [5] and based on the original studies of shock [8,9]. In addition, we chose 2.2 L/min/m^2^ as it was the median of our cohort. Patients with a history of significant intracardiac shunting, left atrial thrombus, left ventricular dysfunction (i.e., low left ventricular ejection fraction), mitral valve stenosis or regurgitation, or enlarged left atrial size were excluded from the study.

### 2.2. CT Acquisition and Measurements

The following image-based exclusion criteria were applied to screen the cases for final analysis: (a) findings that interfere with the normal flow of contrast-enhanced blood from the main pulmonary artery (MPA) to the left atrium (LA) (i.e., significant lung parenchymal pathology, mass effect on LA by large mediastinal mass, etc.); (b) any condition that interferes with the normal flow of opacified blood from the LA to left ventricle (LV) (i.e., mitral apparatus disease, abnormal LV function, significant pericardial disease); (c) significant image artifact that affects the diagnostic quality of the study (i.e., LA streak artifact from high contrast concentration in the superior vena cava and right atrium, significant respiratory motion artifact or gross body motion artifact); (d) suboptimal bolus timing (ascending aortic HU > main PA HU; main PA HU < 250). Out of all the 237 CTAs, 75.1% were performed at our institution.

All in-house pulmonary CTA studies were non-ECG gated and obtained with MDCT scanners in our radiology department (either GE Discovery CT750—Detector configuration 64 × 0.625 or GE Revolution CE- Detector configuration—128 × 0.625; GE Health Care System, Chicago, IL, USA) using a routine clinical protocol [10]. Patient weight-based (small, medium, large) IV contrast medium (Iohexol 350 mgI/mL; GE Healthcare Inc., Chicago, IL, USA) was injected at a rate of 5 mL/s followed by 20 mL saline flush at the same rate in all patients. Scan delay time for optimal enhancement of the pulmonary arteries was determined using bolus tracking at the MPA contrast arrival time trigger threshold of 150 HU + 4 sec post-trigger delay.

For the CTA data collection, all stored images were displayed in the axial plane (2.5–3.75 mm slice thickness) in the mediastinal window setting (WW 400; WL 40) in our Radiology Department PACS System (SECTRA IDS & V24.1.10.5437, SE-111 22 Stockholm, Sweden). Multi-planar reconstruction (MPR) was used for the appropriate placement of a circular ROI (region of interest) of 2 cm diameter, avoiding a partial volume effect. Average Hounsfield unit (HU) measurements were obtained from the MPA, LA, and LV. Care was exercised in the placement of the ROI within the most homogeneously enhanced area of blood, thus avoiding a contrast mixing artifact and streak artifact from the adjacent dense contrast bolus and osseous structures. Within LA, this was the most inferior aspect away from pulmonary veins, just proximal to the mitral valve plane; within LV, it was just distal to the mitral valve plane, avoiding the mitral valve apparatus. To evaluate intra-observer and inter-observer reliability, HU densities were measured twice by two independent radiologists (CD 20 years’ experience in cardiovascular imaging and PN 3rd year Radiology Resident) who were blinded to the remainder of the clinical and hemodynamic data as well as the other radiologist’s measurement.

### 2.3. Statistical Analysis

Data were analyzed using SPSS Statistics (version 29, IBM Corp, New York, NY, USA). Continuous variables were compared between groups using 1-way ANOVA with Tukey’s posthoc, significant difference test, or an unpaired Student’s t-test, as appropriate. Categorical variables were compared between cases and control groups using Pearson’s χ^2^ test; where *n* < 5, Fisher’s exact test was used. A *p* < 0.05 was considered statistically significant, with Bonferroni correction applied as appropriate to control for multiple comparisons. We then calculated HU-Δ by subtracting the HU value of the main PA and LA, and the PA and the LV; HU-Δ were compared between patients with reduced CI and normal CI, as well as the fractional reduction of the HU gradient [(HU-Δ PA-LA/HU PA) × 100] across the PA-LA and the fractional reduction [(HU-Δ PA-LV/HU PA) × 100] across the PA-LV. Interobserver agreement was assessed using Fleiss’ kappa statistics (κ < 0.20, poor agreement; κ = 0.21–0.40, fair agreement; κ = 0.41–0.60, moderate agreement; κ = 0.61–0.80, good agreement; κ = 0.81–1.00, very good agreement). We constructed receiver operating characteristics (ROC) for each variable and used Youden’s J statistic to select a cut-off of the maximized sensitivity and specificity to detect low CI. The study was approved by the Institutional Review Board at TUH (IRB record number 27520).

## 3. Results

### 3.1. Basic Demographics, Comorbidities, and Hemodynamics of the CTEPH Cohort

Of the 237 patients, 119 (50.2%) were female, 126 (53.2%) were White, and 87 (36.7%) were Black. Table 1 includes basic demographics and comorbidities of the CTEPH cohort, with differences between the group with normal versus low CI. Patients with normal CI we predominantly female (65.9% vs. 40.9%, *p* < 0.001) when compared to low CI. There were no differences in age, BMI, WHO FC, comorbidities, or use of PH medications between the groups with normal or low CI.

Hemodynamics were mean (SD) right atrial pressure (RAP) of 10.9 ± 5.1 mmHg, mean pulmonary artery pressure (mPAP) of 45.4 ± 11.2 mmHg, mean pulmonary capillary wedge pressure of 11.8 ± 4.7 mmHg, mean pulmonary vascular resistance (PVR) of 9.2 ± 4.4 Wood unit (WU), mean CI of 2.05 ± 0.48 L/min/m^2^.

### 3.2. Hounsfield Units and Gradients

**Figure 1:**
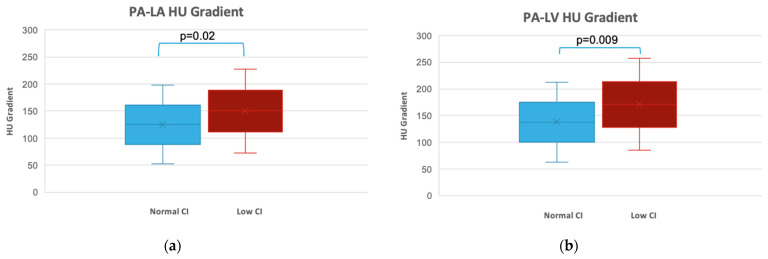
Hounsfield Unit (HU) gradient created by the transit of IV contrast through the heart and lung vascular bed correlates with cardiac index (CI): (**a**) median MPA-LA HU gradient for patients with normal or low cardiac index (*p* = 0.02); (**b**) median MPA-LV HU gradient for patients with normal or low cardiac index (*p* = 0.009).

Mean PA HU and LA HU were 391.1 ± 113.6 and 251.6 ± 81, respectively. Patients with low CI had a higher mean HU-Δ in the PA-LA (148.9 ± 78.4 vs. 124.5 ± 77.2, *p* = 0.02) and PA-LV (170.7 ± 87 vs. 140 ± 82, *p* = 0.009) (Figure 1).

**Figure 2:**
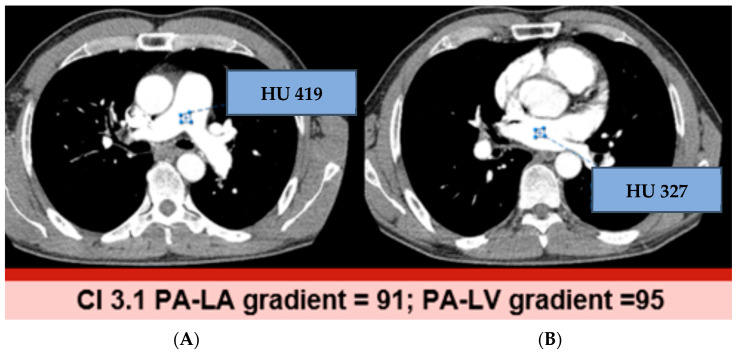
CTEPH case with normal cardiac index. Post contrast CT axial image at the level of MPA with ROI in the MPA (**A**) and LA (**B**). The MPA-LA gradient was 91, which is below the cut-off of 102 that correlates with normal cardiac index (≤102 implies normal CI).

**Figure 3:**
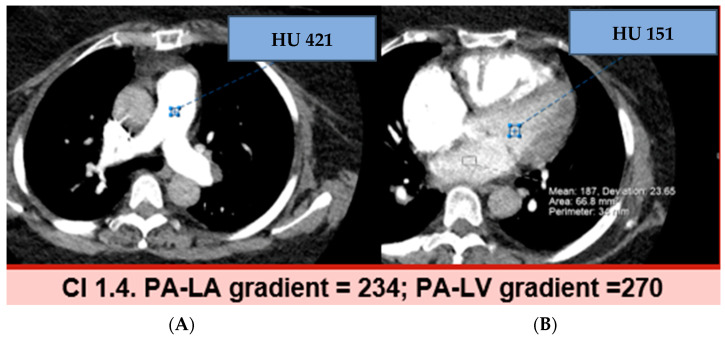
CTEPH case with low cardiac index. Post contrast CT axial image at the level of MPA with ROI in the MPA (**A**) and LV (**B**). The MPA-LV gradient was 270, which is above the cut off of 115 that correlates with low cardiac index (≥115 implies low CI).

Figure 2 and Figure 3 show examples of normal and elevated HU-Δ between PA-LA and PA-LV. The HU-Δ was found to be higher in reduced CI (less blood flow from pulmonary circulation to left heart) than in normal CI. Thus, a possible association was established between CTA-derived parameters and low CI in CTEPH patients. CTA-derived HU-Δ can be used to assess cardiac function in this population.

A Pearson correlation was run to determine the relationship between Δ HU MPA-LA and CI. There was a negative correlation between Δ HU MPA-LA and CI, which was statistically significant (r = −0.140, 95% CI −0.263 to −0.013, *n* = 237, *p* = 0.03). There was a negative correlation between Δ HU MPA-LV and CI, which was statistically significant (r = −0.148, 95% CI −0.271 to −0.02, *n* = 234, *p* = 0.02).

**Figure 4:**
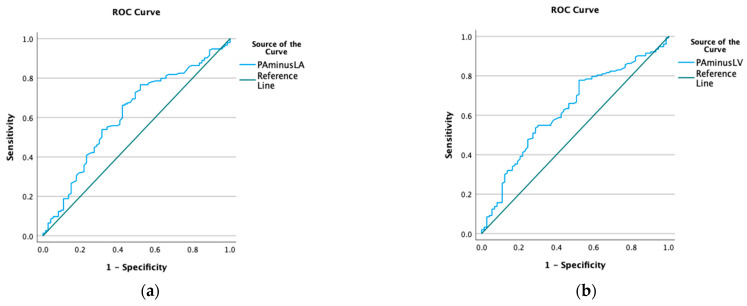
ROC to detect low cardiac index (CI): (**a**) ROC to detect low CI with PA-LA gradient; (**b**) ROC to detect low CI with PA-LV gradient.

A HU-Δ PA-LA of 118 had a sensitivity and specificity of 75.6% and 77%, respectively, to detect low CI (AUC 0.61, *p* = 0.003). (Figure 4) A HU-Δ PA-LV of 156 had a sensitivity and specificity of 77% and 53%, respectively, to detect low CI (AUC 0.62, *p* = 0.001). Supplemental Tables S1 and S2 have different sensitivities and specificities for a CI cut-off less than 2 and 2.5 L/min/m^2^.

### 3.3. Fractional Reduction of HU

**Figure 5:**
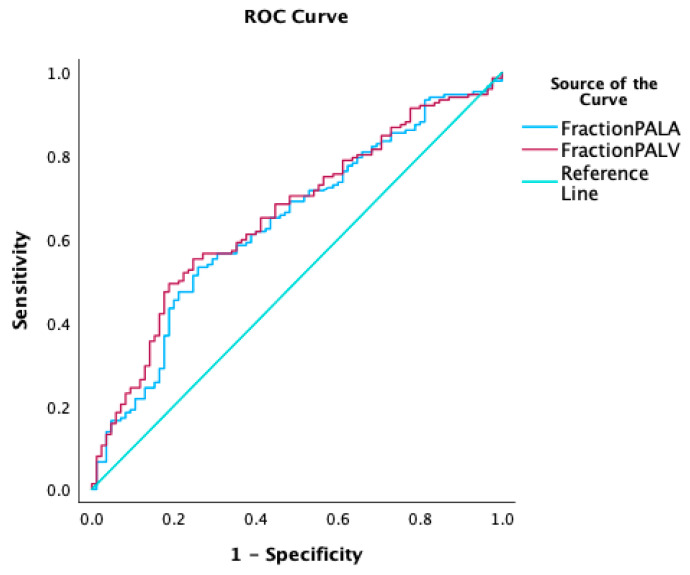
ROC to detect low cardiac index (CI): ROC to detect low CI with the fractional reduction of the PA-LA gradient; ROC to detect low CI with the fractional reduction of the PA-LV gradient.

The fractional reduction of HU (fractional Δ HU) was calculated by normalizing the Δ HU to HU MPA [(Δ HU MPA-LA/HU MPA) × 100]. The average fractional reduction across the PA-LA was 34.8 ± 14.7% and the fractional reduction [(HU-Δ PA-LV/HU PA) × 100] across the PA-LV was 39.6 ± 15.4%. A Pearson correlation was run to determine the relationship between fractional reduction Δ HU MPA-LA or fractional reduction Δ HU MPA-LV and CI. There was a negative correlation between fractional reduction Δ HU MPA-LA and CI, which was statistically significant (r = −0.233, 95% CI −0.35 to −0.11, *n* = 237, *p* < 0.001). There was a negative correlation between fractional reduction Δ HU MPA-LV and CI, which was statistically significant (r = −0.253, 95% CI −0.36 to −0.13, *n* = 237, *p* < 0.001). A fractional reduction of HU-Δ PA-LA of 35% had a sensitivity and specificity of 79% and 53%, respectively, to detect low CI (AUC 0.65, *p* < 0.001). (Figure 5) A fractional reduction score of the HU-Δ PA-LV of 40% had a sensitivity and specificity of 80% and 55%, respectively, to detect low CI (AUC 0.65, *p* < 0.001). Supplemental Tables S1 and S2 have different sensitivities and specificities for a CI cut-off less than 2 and 2.5 L/min/m^2^.

### 3.4. Pulmonary Vein Sign

The pulmonary vein sign was characterized by pulmonary venous flow heterogeneity in the contrast medium filling in the presence of CTEPH [11]. It was seen in 71% of the CTAs evaluated.

### 3.5. Interobserver Variability

Two radiologists independently collected the HU in the main PA, LA, and LV on all patients. The results of the interrater observer coefficient for HU measurements were assessed with Cohen’s weighted kappa analysis. For the HU PA measurement, the Kappa was 0.9 (0.80–0.99 is considered outstanding agreement) with *p* < 0.001, 95% CI 0.86–0.95; for the HU LA measurement, the kappa was 0.85 with *p* < 0.001, 95% CI 0.81–0.91; and for the HU LV measurement, the kappa was 0.89 with *p* < 0.001, 95% CI 0.85–0.93. These measures of agreement are all statistically significant and suggestive of outstanding agreement.

## 4. Discussion

A possible association has been established between pulmonary CTA-derived parameters and low CI in patients with CTEPH. We were to determine that a low CI can be identified by CTA with a fractional reduction of HU between an MPA-LA of 35% and MPA-LV of 40% with a sensitivity of 79 and 80%, respectively. Several factors contribute to the downstream blood enhancement after the IV administration of the contrast medium, such as patient variables (i.e., body weight, cardiac output, venous access), contrast injection variables (i.e., flow rate and concentration, duration of injection, contrast volume), and scanner variables (i.e., scan duration, scan delay, exposure factors) [7]. The most important factors during the first pass blood pool enhancement are the patient body mass, rate of contrast delivery, and the cardiac output.

Soon after IV contrast medium injection, it is diluted by the blood pool and the contrast bolus disperses or widens as it transits downstream through the cardiopulmonary circulatory system. Considering the contrast delivery at the MPA as the ‘system input function’ and the measurement of the CT HU in the LA/LV as the ‘system output function,’ the cardiopulmonary circulatory ‘system function (CI)’ can be reasonably and safely deduced by applying a Linear Time Invariant system model [12]. In our study, the arterial input function is standardized using patient weight-based contrast medium dosing delivered at a constant rate of injection. Since the contrast administration details for 28.2% of our study subjects were not available, we calculated fractional Δ HU (Δ HU normalized to the MPA HU) as our output function, instead of simply relying upon absolute Δ HU between MPA and LA/LV. Given the standardized input function, the output function (fractional Δ HU) itself represents the system function, in our case the CI [13]. In essence, the HU-Δ reflects the downstream mixing and dispersion of IV contrast through the heart and pulmonary vessels, as influenced by the CI.

The results of this retrospective study involving 248 patients with CTEPH shed light on the potential role of CTA-derived HU-Δ in assessing cardiac function in this population. The findings indicated that higher HU-Δ between the PA-LA and PA-LV regions were associated with low CI in patients with CTEPH. Additionally, low CI was demonstrated in CTEPH cases in which a fractional score of HU between the PA-LA was ≥35% or between the PA-LV was ≥40%.

Chronic occlusion or narrowing of the pulmonary vascular bed can lead to abnormal flow patterns compromising the arterial flow, affecting resistance, increasing the right ventricular afterload, and leading to a reduction in the stroke volume and, consequently, the CI. Over time, despite hypertrophy and remodeling of the right ventricle, the pulmonary arterial obstruction causing pulmonary vascular resistance elevation will continue to impair the stroke volume. Reduction in the right ventricular stroke volume will result in decreased venous return and cardiac output, with right ventricular and right atrial enlargement and pericardial constraint leading to LA underfilling and reduction in LV preload.

These results have important clinical implications for risk stratification and the management of CTEPH patients. The non-invasive nature of CTA-based HU-Δ measurement makes it a feasible tool for assessing cardiac function in this population. We recognize that the prognosis of patients with CTEPH is related to the severity of PA pressures [14] and preoperative PVR [15,16], but these variables cannot be readily obtained with a CTA of the chest. In CTEPH, the progression of pulmonary vascular disease related to obstruction will lead to right heart failure and consequently decrease CI. By identifying patients with high HU-Δ or overall high fractional scores, physicians can potentially intervene early and implement appropriate treatment strategies to improve outcomes and prevent further deterioration of cardiac function. A high-quality non-ECG gated CT pulmonary angiography is necessary to extrapolate these hemodynamic data. Additional hemodynamic data via CTA can help multidisciplinary CTEPH groups when evaluating technically accessible disease and assessing the risk/benefit ratio of surgery.

However, it is essential to consider the limitations of our study. Firstly, the retrospective nature of the study design introduces inherent biases and limitations in data collection. Prospective studies with larger sample sizes and standardized protocols must validate these findings. Secondly, the study focused on a single system parameter (i.e., CI) and did not consider other potential contributing factors to low CI in CTEPH patients. However, we excluded patients with low left ventricular ejection fraction or valvular disease. Thirdly, we subjectively evaluated the quality of the images included in the study. Even though we had similar image acquisition characteristics for all the in-house CTAs included, 28.2% of the studies were performed outside our institution. The scanner variables are typically expected to be minimal in the current era of widespread use of MDCT with a standard contrast injection protocol. The potential contrast injection-related variables introduced by the outside facility studies are also partly mitigated by using fractional Δ HU instead of absolute Δ HU. Though the scanner variables may represent a minor limitation, it is also a reflection of the strength of the study and how reproducible the findings are in real-world practice. Fourth, we recognize that there is an overlap in the Δ HU in the values obtained in individuals with normal and low CI, as depicted in Figure 1. This is an issue with small sizes, but we have tried to overcome this limitation by providing ROC curve analysis and suggesting a cut-off with the highest sensitivity and specificity. A HU-Δ PA-LA of 118 had a sensitivity and specificity of 75.6% and 77%, respectively, to detect low CI (AUC 0.61, *p* = 0.003) and a HU-Δ PA-LV of 156 had a sensitivity and specificity of 77% and 53%, respectively, to detect low CI (AUC 0.62, *p* = 0.001). Lastly, there was a time difference between the right heart catheterization and the CTA; the median time difference between the studies was 57 days, but given the chronic nature of the disease, there should be less variability in the hemodynamics in that span of time as well, as we avoided any change in medications within the period. Future research should aim to incorporate additional clinical and hemodynamic variables to further enhance the predictive accuracy of HU-Δ in assessing cardiac function.

Furthermore, the study population was limited to patients referred to a specific hospital’s Pulmonary Hypertension, Right Heart Failure, and CTEPH Program. The findings may not be representative of the broader CTEPH population. Conducting multi-center studies involving diverse patient populations would enhance the generalizability of the results.

Future research should also explore the temporal relationship between changes in HU gradients and CI in CTEPH patients. Longitudinal studies could provide valuable insights into the dynamic nature of this relationship and help identify potential prognostic markers for disease progression.

In addition to assessing the diagnostic performance of HU-Δ, further investigation is warranted to evaluate their prognostic value in predicting clinical outcomes such as mortality, response to treatment, and disease progression. This information would be valuable in risk stratification and guiding treatment decisions in CTEPH patients.

## 5. Conclusions

In conclusion, this study demonstrates a possible association between CTA-derived HU-Δ fractional score and low CI in patients with CTEPH. The findings suggest that HU-Δ could serve as a potential non-invasive parameter for risk stratification and management in this population. However, further research is warranted to validate these findings, address the study’s limitations, and explore the potential clinical applications and prognostic value of HU-Δ in CTEPH.

## Figures and Tables

**Table 1:** Basic Demographics and Comorbidities of CTEPH Cohort.

Variable	All (*n* = 237) *n*(%) or Mean ± SD	Normal CI *n* = 88 *n*(%) or Mean ± SD	Low CI *n* = 149 *n*(%) or Mean ± SD	*p* Value
Female	119 (50.2%)	58 (65.9%)	61 (40.9%)	0.001
Race/Ethnicity				
White	126 (53.2%)	46 (52.3%)	80 (53.7%)	0.372
Black	87 (36.7%)	29 (34.1%)	58 (39.5%)	
Latinx	17 (7.2%)	10 (11.4%)	7 (4.7%)	
Age (years)	60 ± 14.5	59 ± 16	60 ± 14	0.776
BMI (kg/m^2^)	30.1 ± 8.1	31.4 ± 7.3	31.2 ± 8.4	0.642
WHO FC				
I	16 (6.8%)	6 (6.8%)	10 (6.7%)	0.607
II	71 (30%)	29 (33%)	42 (28%)	
III	120 (50.6%)	45 (51.1%)	75 (50.3%)	
IV	30 (12.7%)	8 (9.1%)	22 (14.8%)	
Comorbidities				
Atrial fibrillation	32 (13.5%)	14 (17%)	18 (18.5%)	0.358
Diabetes mellitus	45 (19%)	15 (17.4%)	30 (20.1%)	0.613
History of coagulopathy	54 (21.8%)	19 (22.6%)	31 (20.9%)	0.766
History of DVT	121 (51.1%)	42 (51.2%)	79 (53.7%)	0.714
History of PE	185 (78.1%)	63 (74%)	122 (83%)	0.105
Sleep Disorder	55 (23.2%)	14 (16.7%)	41 (27.7%)	0.057
PH medication	82 (23.2%)	34 (38.6%)	48 (32.2%)	0.218

BMI: body mass index; CI: cardiac index; DVT: deep vein thrombosis; FC: functional class; PE: pulmonary embolism; PH: pulmonary hypertension; WHO: World Health Organization; SD: standard deviation.

## Data Availability

Data are contained within the article and Supplementary Materials.

## Supplementary Materials

The following supporting information can be downloaded at: https://www.mdpi.com/article/10.3390/jcdd11090281/s1, Table S1: Differences using a CI cut-off of less than <2 L/min/m^2^; Table S2: Differences using a CI cut-off of less than <2.5 L/min/m^2^.

## References

[1] Humbert M., Kovacs G., Hoeper M.M., Badagliacca R., Berger R.M., Brida M., Carlsen J., Coats A.J., Escribano-Subias P., Ferrari P. (2022). 2022 ESC/ERS Guidelines for the diagnosis and treatment of pulmonary hypertension. Eur. Heart J..

[2] Galiè N., Humbert M., Vachiery J.L., Gibbs S., Lang I., Torbicki A., Simonneau G., Peacock A., Vonk Noordegraaf A., Beghetti M. (2016). 2015 ESC/ERS Guidelines for the diagnosis and treatment of pulmonary hypertension: The Joint Task Force for the Diagnosis and Treatment of Pulmonary Hypertension of the European Society of Cardiology (ESC) and the European Respiratory Society (ERS): Endorsed by: Association for European Paediatric and Congenital Cardiology (AEPC), International Society for Heart and Lung Transplantation (ISHLT). Eur. Heart J..

[3] Mahmud E., Behnamfar O., Ang L., Patel M.P., Poch D., Kim N.H. (2018). Balloon Pulmonary Angioplasty for Chronic Thromboembolic Pulmonary Hypertension. Interv. Cardiol. Clin..

[4] Mahammedi A., Oshmyansky A., Hassoun P.M., Thiemann D.R., Siegelman S.S. (2013). Pulmonary artery measurements in pulmonary hypertension: The role of computed tomography. J. Thorac. Imaging.

[5] Brailovsky Y., Masic D., Allen S., Lakhter V., Bashir R., Forfia P., Bechara C.F., Leya F.S., Lopez J.J., Lewis B.E. (2021). Novel CT-derived parameter is associated with low cardiac index in acute pulmonary embolism. Thromb. Res..

[6] Gopalan D., Riley J.Y.J., Leong K., Alsanjari S., Auger W., Lindholm P. (2023). Computed Tomography Pulmonary Angiography Prediction of Adverse Long-Term Outcomes in Chronic Thromboembolic Pulmonary Hypertension: Correlation with Hemodynamic Measurements Pre- and Post-Pulmonary Endarterectomy. Tomography.

[7] Bae K.T. (2010). Intravenous contrast medium administration and scan timing at CT: Considerations and approaches. Radiology.

[8] Forrester J.S., Diamond G., Chatterjee K., Swan H.J. (1976). Medical therapy of acute myocardial infarction by application of hemodynamic subsets (first of two parts). N. Engl. J. Med..

[9] Nohria A., Tsang S.W., Fang J.C., Lewis E.F., Jarcho J.A., Mudge G.H., Stevenson L.W. (2003). Clinical assessment identifies hemodynamic profiles that predict outcomes in patients admitted with heart failure. J. Am. Coll. Cardiol..

[10] University of Wisconsin-Madison GE CT Protocol Partnership. Protocol Manuals..

[11] Gopalan D., Nordgren-Rogberg A., Le E.P.V., Pavey H., Tarkin J., Nyrén S., Auger W., Lindholm P. (2020). Abnormal Pulmonary Venous Filling: An Adjunct Feature in the Computed Tomography Pulmonary Angiogram Assessment of Chronic Thromboembolic Pulmonary Hypertension. J. Am. Heart Assoc..

[12] Fleischmann D., Kamaya A. (2009). Optimal vascular and parenchymal contrast enhancement: The current state of the art. Radiol. Clin. N. Am..

[13] Parker A. (1990). Image Reconstruction in Radiology.

[14] Riedel M., Stanek V., Widimsky J., Prerovsky I. (1982). Longterm follow-up of patients with pulmonary thromboembolism: Late prognosis and evolution of hemodynamic and respiratory data. Chest.

[15] Jamieson S.W., Kapelanski D.P., Sakakibara N., Manecke G.R., Thistlethwaite P.A., Kerr K.M., Channick R.N., Fedullo P.F., Auger W.R. (2003). Pulmonary endarterectomy: Experience and lessons learned in 1500 cases. Ann. Thorac. Surg..

[16] Mayer E., Jenkins D., Lindner J., D’armini A., Kloek J., Meyns B., Ilkjaer L.B., Klepetko W., Delcroix M., Lang I. (2011). Surgical management and outcome of patients with chronic thromboembolic pulmonary hypertension: Results from an international prospective registry. J. Thorac. Cardiovasc. Surg..

