# Supplementary material for: Novel Computed Tomography Angiography Parameter Is Associated with Low Cardiac Index in Patients with Chronic Thromboembolic Pulmonary Hypertension: A Retrospective Analysis
Source: J Cardiovasc Dev Dis. 2024 Sep 7;11(9):281. doi: 10.3390/jcdd11090281 (PMC11432232; doi:10.3390/jcdd11090281)
Supplement: Supplementary file 1 [file jcdd-11-00281-s001.zip › jcdd-3060475-supplementary.pdf]

Supplemental Table S1: Differences using a CI cut off of less than <2 L/min/m2

	AUC	95% CI	P value	Cut off	Sensitivity	Specificity
HU-Δ MPA-LA HU	0.62	0.55-0.69	<0.001	106.5	78.4	53
HU-Δ MPA-LV HU	0.63	0.56-0.7	<0.001	137	67	52
Fractional reduction HU-Δ PA-LA	0.65	0.58-0.72	<0.001	40.5	48	61
Fractional reduction HU-Δ PA-LV	0.66	0.6-0.74	<0.001	43	54	59

Supplemental Table S2: Differences using a CI cut off of less than <2.5 L/min/m2

	AUC	95% CI	P value	Cut off	Sensitivity	Specificity
HU-Δ MPA-LA HU	0.64	0.54-0.73	0.004	106.5	70	88
HU-Δ MPA-LV HU	0.64	0.55-0.7	0.002	115	73	88
Fractional reduction HU-Δ PA-LA	0.68	0.58-0.76	<0.001	34.8	51	90
Fractional reduction HU-Δ PA-LV	0.69	0.6-0.77	<0.001	34.5	67	89
